# Standardised Parenteral Nutrition

**DOI:** 10.3390/nu5041058

**Published:** 2013-03-28

**Authors:** Karen Simmer, Abhijeet Rakshasbhuvankar, Girish Deshpande

**Affiliations:** 1 Department of Neonatal Paediatrics, King Edward Memorial Hospital for Women and Princess Margaret Hospital for Children, Subiaco, WA 6008, Australia; E-Mail: abhijeet.rakshasbhuvankar@health.wa.gov.au; 2 Centre of Neonatal Research and Education, School of Paediatrics and Child Health, University of Western Australia, Crawley, WA 6009, Australia; 3 Department of Neonatal Paediatrics, Nepean Hospital, Kingswood, NSW 2747, Australia; E-Mail: Girish.Deshpande@swahs.health.nsw.gov.au; 4 Sydney Medical School Nepean, University of Sydney, Sydney, NSW 2747, Australia

**Keywords:** premature, nutrition, standardised parenteral nutrition, individualised parenteral nutrition, total nutrient admixtures, triple-chamber bag

## Abstract

Parenteral nutrition (PN) has become an integral part of clinical management of very low birth weight premature neonates. Traditionally different components of PN are prescribed individually considering requirements of an individual neonate (IPN). More recently, standardised PN formulations (SPN) for preterm neonates have been assessed and may have advantages including better provision of nutrients, less prescription and administration errors, decreased risk of infection, and cost savings. The recent introduction of triple-chamber bag that provides total nutrient admixture for neonates may have additional advantage of decreased risk of contamination and ease of administration.

## 1. Introduction

The placenta is the only source of nutrition for growing fetus during the intrauterine life. Neonates delivered at less than 30 weeks gestation are born at a time of rapid brain and body growth. Abrupt cessation of the placental supply of nutrients at birth makes these premature neonates vulnerable to nutritional deficiencies unless enteral or parenteral nutrition is established rapidly. In very premature neonates enteral feeding is often established slowly and therefore, during this period, nutrients are provided parenterally in the form of parenteral nutrition (PN). Traditionally, different components of PN for neonates are prescribed individually taking into consideration the biochemical, nutritional and physiological status of the neonate. However, standardised PN (SPN) combinations have been evaluated and may have some advantages over the individualised PN (IPN) regimen. 

Very low birth weight (VLBW) neonates have changing physiology and clinical condition during the first few days of life. It is reasonable to think that PN ordered considering unique requirements of a particular newborn infant will be the most appropriate and will give the best possible outcome in terms of biochemical control, nutrient intake and weight gain. However, studies have indicated that most premature neonates tolerate mild to moderate variations in nutritional intake and majority of those can be managed with few sets of standard PN solutions [1,2]. 

## 2. SPN Formulations

SPN formulations are in use in many Neonatal Intensive Care Units (NICUs) across Australia. These PN formulations are available commercially or can be made in-house by hospital pharmacist. 

There were over 60 different neonatal PN formulations supplied by Baxter^®^ across Australia and New Zealand. These PN formulations have a shelf life up to 40 days at 2–8 °C. Collaboration between NICUs has recently greatly reduced the number and cost of commercial formulations (Bolisetty, PSANZ Sydney abstract). 

In our NICU (King Edward Memorial Hospital for Women, Western Australia), SPN has been made by our pharmacist for over a decade. We have formulations with glucose concentrations of 6%, 8%, 10% and 12% and amino acid concentrations of 1, 2 and 3 g/100 mL with standard amount of electrolytes and vitamins. These formulations are made in our hospital pharmacy during the working-hours seven days a week. After-hours for a new patient, we use commercially available SPN (Starter TPN) (Baxter^®^) containing amino acid (1.5 g/100 mL) and glucose (5% or 7.5%) until pharmacy-made PN is available. We start 20% olive oil based lipid emulsion (ClinOleic^®^, Baxter^®^) at a dose of 1 g/kg within 24 h of birth. Olive oil based lipid emulsions have been found to be well tolerated by critically ill and preterm neonates. [3,4,5,6]. Several *in vitro* and animal studies have reported suppression of T-lymphocyte function and impaired bacterial clearance by soybean oil based lipid emulsion compared with minimal effect from olive oil based emulsion. [7]. In addition there are concerns regarding excess of poly-unsaturated fatty acids (PUFAs) and low vitamin E levels in soybean oil based lipid emulsions [8]. Although, the clinical studies have failed to show any short term benefits of using olive oil based emulsions on fatty acid profile and anti-oxidant properties, the fatty acid profile of preterm neonates in Clinoleic group was similar to breast milk fed preterm neonates [4,5,6]. ClinOleic^®^ 20% lipid emulsion contains a mixture of 80% olive oil and 20% soybean oil and is given as a 24 h infusion piggy-backed to the rest of the PN solution containing glucose, amino acids, electrolytes and vitamins. When given 160 mL/kg/day volume, the SPN containing 2 g/100 mL amino acids, 12% glucose and 3 g/kg of lipid emulsion gives 114 Kcal/kg of energy and 3.2 g/kg of proteins. This nutrient intake is consistent with recent ESPGHAN guidelines which recommend energy intake of 110–120 Kcal/kg and protein intake of 3–4 g/kg body weight in premature infants [9]. 

## 3. PN in Premature Neonates

One of the most significant therapeutic advances of 20th century came when Dudrick *et al.* demonstrated practical method of providing total nutrition intravenously [10]. Initial need for intravenous nutrition was perceived in the post-operative adult patients who were kept fasting for extended period of time. It was seen that under-nutrition in these patients was associated with increased morbidity and mortality. Over the last 60 years, the indications, objectives, constituents and methods of administration of PN have evolved. 

Nutrition of the newborn infant, previously often a neglected issue, has been gaining increasing importance in acute clinical management. It is becoming clear that early nutrition in the critical period plays an important role in the long-term health and neuro-development. Experimental studies in animals have shown that nutrition in the critical period of life can affect brain structure and function irreversibly [11,12]. Postnatal nutrition in rat male pups was shown to affect dendritic branching in certain locations of rat brain, important in regulating attention status and the integration of motor and sensory activity, and this effect persisted in spite of later correction of nutritional deficits [13]. The potential vulnerability of the human brain to early suboptimal nutrition was reported by Lucas *et al*. The authors in their prospective randomised blinded trial observed that better nutrition of preterm newborn male infants was associated with a lower incidence of cerebral palsy and higher IQ scores at 7–8 years of age [14]. It is postulated that post-natal under-nutrition at a sensitive or critical period of brain growth or maturation influences the programming pathways in the brain permanently, influencing the cognitive performance later in the life [15,16].

Post-natal growth retardation (PNGR) is common in the VLBW premature infants [17,18,19]. With increasing survival of premature infants, research is focused on decreasing the morbidities associated with premature birth. Provision of adequate nutrition soon after birth to match the fetal accretion rate, is important to reduce PNGR and associated impaired neuro-developmental, metabolic disorders and persistent short stature [20,21,22,23,24,25]. Thus evidence indicates that it may be critical to establish adequate nutrient supply soon after birth to prevent long term adverse effects of inadequate nutrition. 

## 4. SPN *vs*. IPN

The studies comparing nutrient intakes during SPN and IPN are mostly non-randomised cohort studies, some favouring IPN while others SPN (Table 1) [26,27,28,29,30,31]. The only randomized controlled trial (RCT) comparing IPN with SPN enrolled only a small number of neonates [27]. The authors assigned 28 neonates requiring PN in either SPN or IPN group. They found that IPN led to better intake of calories, protein and lipids; and resulted in improved weight gain as compared with SPN. Glucose was the most common component of PN, which needed to be adjusted in SPN group. The adjustment was in the form of addition of dextrose to the SPN, which corrected the glucose homeostasis but diluted the amino acids resulting in decreased protein intake in SPN group [27]. Similarly, increased nutrient intakes with the use of IPN were also seen in a retrospective observational study by Mulchie *et al.* [26].

**Table 1:** Standardised *vs*. individualised PN—studies in neonates.

Author [ref.] (Year)	Location	Number of study subjects	Age group	Study design	Summary of results
Mulchie [26] (1979)	Paediatric Hospital	12	<36 days, mean GA 35 weeks	Cohort	Mean weight gain in SPN group was 4 g/day *vs*. 17 g/day in IPN group.
Dice [27] (1981)	NICU	28	Mean GA 31 weeks	RCT	IPN group received significantly higher energy and protein intake and had significantly higher weight gain (11.8 *vs*. 4.9 g/day).
Yeung [28] (2003)	NICU	31 in 1999/2000 (IPN) *vs*. 27 in 2000/1 (SPN)	GA < 33 weeks	Cohort	SPN group received significantly more proteins each day; and more calcium and phosphate on day 3. SPN was associated with significant cost reduction.
Lenclen [29] (2006)	NICU	20 in 2001 (IPN) *vs*. 20 in 2003 (SPN)	GA < 32 weeks	Cohort	On day 3, intakes of carbohydrates and AA were higher; and calcium phosphate intakes were better balanced in SPN group.
Smolkin [30] (2010)	NICU	70 in 2000–2001 (SPN) *vs*. 70 in 2006–2007 (IPN)	VLBW newborn infants	Cohort	IPN group received significantly higher daily intake of glucose, protein and fat; and achieved full enteral feeds faster.
Iacobelli [31] (2010)	NICU	40 in 2006 (IPN) *vs*. 67 in 2006–2007 (SPN)	GA < 33 weeks	Cohort	SPN group received significantly more glucose, AA, lipids, sodium and magnesium. SPN was associated with significantly reduced weight loss on day 7.

GA: Gestational age at birth; SPN: Standardised parenteral nutrition; IPN: Individualised parenteral nutrition; NICU: Neonatal Intensive Care Unit; RCT: Randomised controlled trial; AA: Amino acids.

Smolkin *et al.* in a retrospective observational study involving 140 VLBW neonates reported that IPN was associated with significantly greater weight gain during the first month of life, greater discharge weight, shorter duration of PN requirement and more electrolyte stability [30]. The authors attributed the difference to the “richer” nutrition contents of IPN as compared with SPN. A weakness of the study was a long interval of six years between the two study periods, SPN cohort from year 2000 to 2001 and IPN cohort from year 2006 to 2007, with the possibility of change in the clinical care contributing to the outcome [30].

Other studies have reported favourable outcome with SPN when compared with IPN [28,29,31]. Lenclen *et al.* in their observational study reported that SPN provided higher early intakes of amino acids and glucose, and a better calcium phosphate ratio [29]. Improvement in nutrient intakes obtained by using SPN was because of less deviation from the protocol and earlier start of PN after birth [29]. Yeung *et al.* in their retrospective observational study in neonates <33 weeks gestational age found a similar benefit of improved nutrient intake with SPN [28]. The authors found that when compared with IPN, SPN was associated with 35% less cumulative deficit in protein intake by the end of first week and, higher calcium and phosphate intakes. They did not find any clinical advantage of improved biochemical control with the IPN regimen [28]. Similar findings of higher protein and energy intakes without an increased risk of metabolic disturbances were also reported by Iacobelli *et al.* in their prospective observational study involving 107 neonates born at <33 weeks gestation [31].

## 5. Early and Aggressive Nutrition

It is a well-known fact that preterm neonates have limited energy reserves at birth and adequate provision of calories and protein to match intrauterine accretion rate soon after birth is required to prevent catabolic state [32]. The practice is often referred as “Aggressive” nutrition. One advantage of SPN is the ready availability in NICUs enabling initiation of the PN within a hour of birth [29,33]. IPN on the other hand is often not available especially afterhours. Starting PN within hours of birth has been found to be associated with positive nitrogen balance and calorie intake without increasing the risk of metabolic complications [34,35]. Aggressive intakes of amino acids in the range of 2.5–3 g/kg/day starting on day one of life are well tolerated. [35,36]. The use of such aggressive nutrient intake is associated with increased protein accretion, decreased PNGR, better potassium homeostasis with decreased incidence of non-oliguric hyperkalemia, and decreased incidence of hyperglycemia by stimulation of endogenous insulin secretion. [31,37,38,39,40]. In a recent prospective observational study by Senterre and Rigo, ready-to-use SPN formulation was effective to limit the cumulative nutritional deficit and PNGR by providing early and aggressive nutrition to preterm neonates less than 1250 g [40,41].

## 6. Electrolytes Homeostasis

Renal and liver functions in the premature neonates continue to develop after birth. In addition, fluid and electrolyte balance is affected by extra-renal systems, illness, medications and interventions [42]. The fixed electrolyte contents of SPN may not be tolerated well by the sick premature neonates. However, Devlieger *et al.* proposed that the premature neonates are capable, within certain limits, of appropriate homeostasis as early as the first week of life and hence may be managed with few combinations of standard PN formulations [43]. The authors found that SPN formulations were sufficient to manage most of the VLBW neonates without significant electrolyte disturbances [43]. An advantage of IPN is the ability to make changes in the electrolyte composition of the PN to suit the neonates. However, there is often a delay (up to 24 h) between the blood sampling and the administration of the TPN making changes in the PN based on these blood results far from ideal and possibly at times deleterious to biochemical homeostasis [43]. 

## 7. Errors/Variations

PN is one of the most complex medications with more than 50 constituents. There are multiple stages in the process of PN management where errors can occur: prescribing, transcription, preparation, and administration [44]. In an observational study in the adult patients receiving PN in an academic teaching general hospital, Sacks *et al.* found that out of 4730 PN prescriptions, 1.6% were associated with an error. Most of the errors occurred in transcription and administration process [45,46]. Studies have reported that IPN is associated with a high incidence of medical errors and protocol deviations [47] while SPN formulations have been associated with significantly less prescription errors in adult patients [48]. We did not find any study describing frequency of prescription errors while writing paediatric or neonatal PN prescriptions. It has been reported that individualized PN forms are oriented towards those who compound PN formulations and have been designed for ease of pharmacy usage; whereas, standardized PN forms are oriented toward ease of physician use. In a study by Mitchell *et al.* in adult patients, there was a substantial decrease in the prescription error and thus, a decrease in metabolic complications, with the use of standardized PN forms [49]. 

As use of PN has been increasing in the NICUs there is a need to establish prescription standards. Physicians, from attending staff to resident staff, differ greatly in their levels of nutrition education, especially in their familiarity with ordering the parenteral solutions. IPN puts more responsibility on the prescribing physicians to ensure adequate nutrient delivery [48]. The quality of IPN depends upon knowledge and attitude of the person ordering the PN. Optimal PN needs to be adequately prescribed and requires well trained physicians [2]. Studies have reported lack of education and knowledge regarding nutritional needs of neonates among doctors responsible for prescribing IPN [49]. SPN may provide the physician with a theoretically optimal starting point, and help standardise and optimise the PN prescription practices. 

## 8. Risk of Infection

Administration of TPN, especially when prolonged, is associated with increased risk of late onset sepsis [50,51]. Most of the blood-stream infections (BSI) related to PN are caused by contamination of the device used for percutaneous vascular access; however, the fluid administered through the device also can become contaminated and cause BSI [52]. Various outbreaks of hospital-acquired infections have been reported through administration of contaminated PN because of lapses in sterility during compounding PN at hospital pharmacy [53]. SPN especially when commercially prepared may decrease chances of contamination of PN [54].

## 9. Cost

PN therapy is relatively expensive therapy, especially when personnel cost for patient monitoring, catheter care, and solution compounding are added to material cost. TPN compounding requires special, expensive equipment and infrastructure. Increasing use of TPN in relatively smaller units has created administrative and clinical challenges for hospital pharmacies. SPN may be the solution for such small hospitals if found to be well tolerated by neonates. SPN decreases processing and compounding time; and material cost for PN [48]. These resources can be utilized for other purposes in resource-restricted settings. SPN was associated with 30% reduction in the cost of PN in a study by Yeung *et al.* [28]. SPN decreased solution wastage, labor and material costs, and inventory-holding costs, resulting in a 56% decrease in annual TPN related expenditures [55]. Similar findings of reduced cost with SPN were also reported by Roberts *et al.* (1981), and Petros *et al.* (1986) [48,56].

## 10. 3-in-1 PN (Total Nutrient Admixtures)

Total nutrient admixture (TNA), single mixture of all the components of PN, has been found to be safe and well tolerated in adults [48,57,58,59,60]. Perceived advantages of TNA system in adults include shorter time required for daily administration of PN resulting in decrease in nursing time and cost for patient care; and decrease in the risk of formula and vascular access contamination related to reduction of preparation steps, fewer solution containers, decreased violations of the central catheter, avoidance of piggybacking and the inadvertent dislodgement of the additional infusion tubing [55,56,58,61]. However, there is little data regarding its use in neonatal population.

Intravenous fat emulsions (IVFE) are found to be associated with increased risk for bacterial and fungal sepsis [61,62,63]. IVFE poses a major risk for sepsis in premature infants because of its favourable pH for growth of bacteria and fungi. In addition, lack of small volume containers, from manufacturers, appropriate for premature infants requires it to be repackaged in the pharmacy. Repackaging of intravenous fat emulsions even using aseptic technique under International Organisation for Standardization (ISO) class 5 conditions was associated with 1.7% rate of contamination [64,65]. 3-in-1 PN preparations may provide some protective effect for lipid emulsion associated BSI.

**Figure 1:**
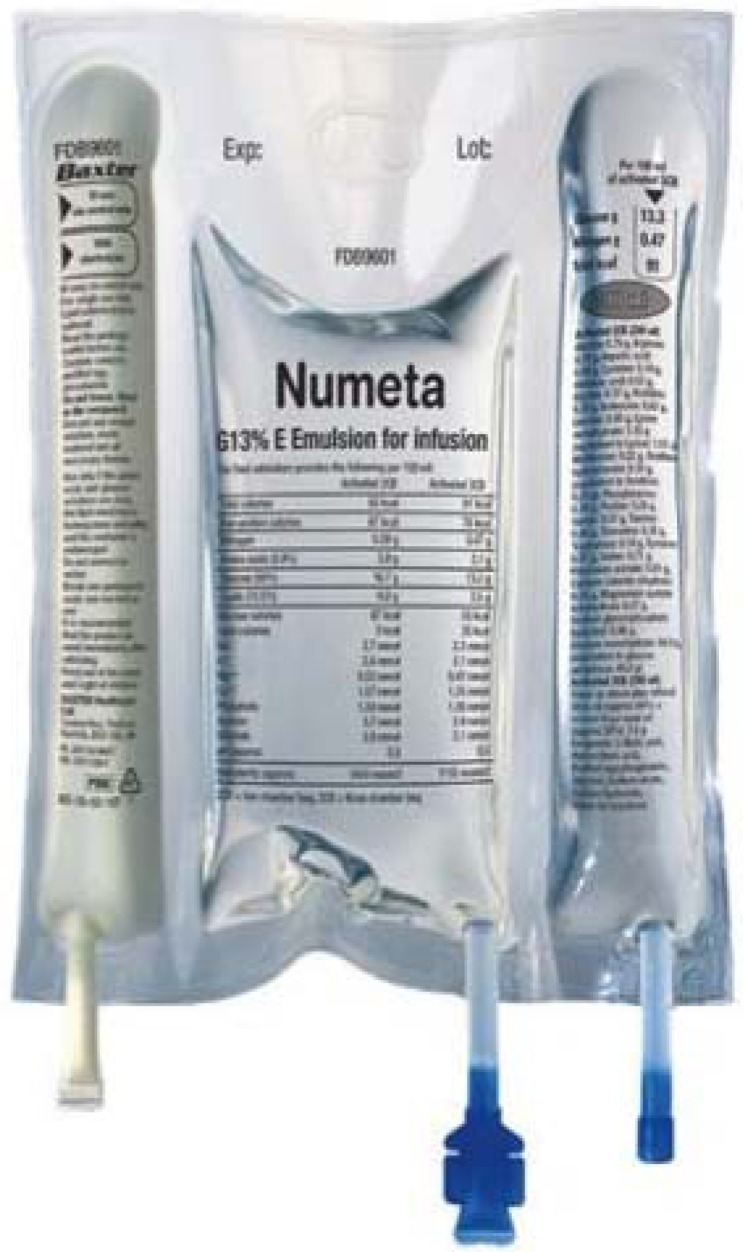
Numeta^®^ (Baxter^®^ International Inc.) triple chamber bag for neonatal use.

Recently, 3-in-1 PN has become available as a triple chamber system (Numeta^®^, Baxter International Inc.); (Figure 1) for use in paediatric and neonatal population. The product has been licensed in 16 European countries; however, is not yet available in USA and Australia. The bag contains a glucose solution (13%, 16% or 19%), a paediatric amino acid solution (Primene^®^, Baxter^®^) with electrolytes and olive oil based lipid emulsion (ClinOleic^®^, Baxter^®^) in different compartments separated by seal, which can be broken to mix the components just before administration. Having the major components of PN separate prolongs its shelf life. Shelf life of Numeta^®^ triple chamber PN bag is 18 months. It remains stable for 48 h at 30 °C after mixing of the components [66]. The bag also gives a choice of withholding lipids, by keeping the seal unbroken between the lipid emulsion and the rest of the PN compartment. The preparation does not contain trace elements and vitamins, which are added into the final mixture separately as per clinical needs of the patients. The product has some unique components (ClinOleic, Primene and sodium glycerophosphate hydrated) which are not commercially available in USA. 

A recent study by Rigo *et al.* evaluated safety and feasibility of the triple-chamber bag in 97 premature neonates [67]. The triple-chamber bag PN was administered for a minimum of 5 days and maximum of 10 days. The authors found that PN administration using commercially available triple chamber bag was safe and practical in premature neonates [67]. Additional supplementation, mainly of sodium, was needed to be performed in 45% infusion days, primarily using a Y-line; while additions directly to the triple chamber bag occurred on 21% infusion days in 37% infants. The authors found the nutritional intake in the range of “aggressive” nutrition recommendation and weight gain of 22 g/kg/day after the first week of life in their infants suggesting use of triple-chamber bag may reduce post-natal growth deficit. Absence of a control group is an important limitation, as mentioned by authors, of this study [67,68].

## 11. Conclusion

Provision of adequate nutrition without causing biochemical derangement is an integral part of neonatal intensive care management. SPN may have advantages over IPN with respect to higher nutrient intake and weight gain; and less prescription errors and cost; without causing significant biochemical disturbance. Recently introduced ready-to-use all-in-one PN preparations for neonatal use may have additional advantages of ease of administration and decreased risk of infection. 

## 12. Future Directions

Well-controlled randomised controlled trials are needed to establish the role of SPN in the neonatal population including the safety, efficacy and feasibility of triple chamber bag TPN in preterm infants. The studies ideally need to evaluate not only the short term outcomes but also the long term outcomes—such as growth, neuro-developmental outcome and association with metabolic disorders—as rapid weight gain in neonatal period may be associated with increased risk of insulin resistance and obesity in adulthood [22].

**Key Points**
- SPN may be well tolerated by very premature newborn infants without significant biochemical disturbances.
- SPN may have advantages over the IPN in terms of less prescription and administration errors, decreased risk of infection, and cost savings.
- Triple chamber bag for TPN will provide an additional alternative for preterm neonates; however more well-controlled RCTs are needed measuring short term and long term outcomes.
